# Medical Opinion and Movement

**Published:** 1907-10-12

**Authors:** 


### Medical Opinion and Movement.

The National Temperance League has arranged for a series of important scientific researches into the action of alcohol upon mental and muscular efficiency, and upon the blood in relation to its power of resistance to disease. The League has had the valuable assistance of a science and education committee, composed of prominent members of the medical profession specially interested in the subject, such as Sir Thomas Barlow, Sir Victor Horsley, Professor Sims Woodhead, and others. Several different lines of research have been arranged. The capacity for muscular work with graduated doses of alcohol will be tested and compared with the same work without alcohol. Similarly tests of capacity for mental work will be carried out in the same way. The effects of alcoholic beverages in ordinary use will also be compared with those of pure ethylic alcohol. These researches should prove highly interesting, and should afford useful material for the solution of the many problems involved in the use and abuse of alcohol as a beverage and as a therapeutic drug.

An interesting case of tetanus successfully treated by the intraspinal injection of a solution of magnesium sulphate has been lately reported by Dr. G. C. Robinson in the *Journal of the American Medical Association*. The patient was a boy aged 11, who developed pain and stiffness of the jaws, and within a week of the onset general rigidity of the muscles had set in. The condition became acute, with general spasms every few minutes. Lumbar puncture was performed, and clear intraspinal fluid rose in the manometer to 235 mm. Three cubic centimetres of a 25 per cent. solution of magnesium sulphate were injected. In a short time the rigidity passed off completely, but returned within twenty-four hours. The injection was repeated, with the same result. Complete relaxation now lasted for a few days, and then rigidity gradually returned. A third injection, however, effected a complete cure. The idea of using magnesium sulphate injections in cases of tetanus was originally conceived by Meltzer. He experimented on animals with subcutaneous injections of the salt, and found that they produced prolonged anæsthesia with muscular relaxation and abolition of reflexes. Intravenous injections depressed the medullary centres for respiration and deglutition, but did not affect the circulatory mechanism. In monkeys intraspinal injections were followed by complete surgical anæsthesia. A few abdominal operations have been performed after intraspinal injections of a 25 per cent. solution of the salt. These produced anæsthesia with muscular relaxation, loss of tendon reflexes and slowing of respiration. In six out of fourteen cases the patients lost consciousness.

As a result of the amended law in regard to compulsory vaccination, the Local Government Board has issued a new Vaccination Order, in accordance with which every informant of a birth on and after September 1 will receive, in addition to a copy of the revised "Notice of Requirement of Vaccination," a copy of the "Form of Declaration" of conscientious objection. Parents will in this way be saved all possible trouble in their desire to avoid the vaccination of their children. They will have the two forms to hand. To get the one signed they must call in the doctor, and in addition they must face the possibility of one or two restless nights with the baby. The alternative course is to take the "Form of Declaration" to the nearest commissioner of oaths. It seems probable that the average individual, who cares little for possible eventualities, will adopt the latter course as the simplest and least inconvenient, even though he is not imbued with any anti-vaccination prejudices. But it is not likely that the anti-vaccination propagandists will allow such an opportunity to pass. They have the weakness of humanity on their side, and will doubtless assist the ignorant masses to follow the easier course. We may underestimate the intelligence of the community. But the ordinary layman cannot be expected to know what he is not taught, and we know of no serious efforts that have been made to instruct him in the actual facts in regard to vaccination and its prophylaxis against small-pox. Some such effort will, we fear, be seriously required, if we are to prevent the calamity which is bound to fall upon a community unprotected against small-pox.

The administration of antitoxin as a prophylactic against diphtheria in outbreaks of the disease has been much favoured of late by the profession, though the conditions under which it has been employed make it difficult to estimate the measure of its efficacy. In discussing the question, Dr. Owen H. Peters, of Nottingham, contrasts the methods used to combat two outbreaks of diphtheria at Nottingham during 1906. In the first, which occurred at the General Hospital, a wholesale injection of antitoxin, together with swabbing and bacteriological examination of the throats of the staff, was carried out. Twenty-one were found to be infected with the Klebs-Loeffler bacillus and were promptly isolated, and of these seven developed the disease, in spite of the antitoxin, within three weeks of the injections. As a result of these measures no further cases occurred. In the second outbreak, amongst school children at Dunkirk, an isolated corner of the borough, the throats of 2,000 children were examined and twenty were found to harbour the bacillus. These "carriers" were isolated and the schools closed. Altogether about thirty "suspects" were received into hospital. No antitoxin was administered, and only three developed the disease. In this instance also the outbreak was stopped. These facts strongly support Dr. Peters' contention that the essential measures of prophylaxis consists in the thorough examination of all throats of those exposed to infection and the isolation of those found to be "carriers" of the bacillus. The administration of antitoxin to cases so isolated may prevent the development of the disease, but it should not be relied upon as a primary means of preventing a spread of the disease.

